# Neighborhood Disadvantage and Neural Correlates of Threat and Reward Processing in Survivors of Recent Trauma

**DOI:** 10.1001/jamanetworkopen.2023.34483

**Published:** 2023-09-18

**Authors:** E. Kate Webb, Timothy D. Ely, Grace E. Rowland, Lauren A. M. Lebois, Sanne J. H. van Rooij, Steven E. Bruce, Tanja Jovanovic, Stacey L. House, Francesca L. Beaudoin, Xinming An, Thomas C. Neylan, Gari D. Clifford, Sarah D. Linnstaedt, Laura T. Germine, Kenneth A. Bollen, Scott L. Rauch, John P. Haran, Alan B. Storrow, Christopher Lewandowski, Paul I. Musey, Phyllis L. Hendry, Sophia Sheikh, Christopher W. Jones, Brittany E. Punches, Robert A. Swor, Jose L. Pascual, Mark J. Seamon, Elizabeth M. Datner, Claire Pearson, David A. Peak, Roland C. Merchant, Robert M. Domeier, Niels K. Rathlev, Paulina Sergot, Leon D. Sanchez, Ronald C. Kessler, Karestan C. Koenen, Samuel A. McLean, Jennifer S. Stevens, Kerry J. Ressler, Nathaniel G. Harnett

**Affiliations:** 1 Division of Depression and Anxiety, McLean Hospital, Belmont, Massachusetts; 2 Department of Psychiatry, Harvard Medical School, Boston, Massachusetts; 3 Department of Psychiatry and Behavioral Sciences, Emory University School of Medicine, Atlanta, Georgia; 4 Department of Psychological Sciences, University of Missouri-St Louis; 5 Department of Psychiatry and Behavioral Neurosciences, Wayne State University, Detroit, Michigan; 6 Department of Emergency Medicine, Washington University School of Medicine, St Louis, Missouri; 7 Department of Epidemiology, Brown University, Providence, Rhode Island; 8 Department of Emergency Medicine, Brown University, Providence, Rhode Island; 9 Institute for Trauma Recovery, Department of Anesthesiology, University of North Carolina at Chapel Hill; 10 Department of Psychiatry, University of California, San Francisco; 11 Department Neurology, University of California, San Francisco; 12 Department of Biomedical Informatics, Emory University School of Medicine, Atlanta, Georgia; 13 Department of Biomedical Engineering, Georgia Institute of Technology and Emory University, Atlanta; 14 The Many Brains Project, Belmont, Massachusetts; 15 Institute for Technology in Psychiatry, Harvard Medical School, Boston, Massachusetts; 16 Department of Psychology and Neuroscience, University of North Carolina at Chapel Hill; 17 Department of Sociology, University of North Carolina at Chapel Hill; 18 Department of Psychiatry, McLean Hospital, Belmont, Massachusetts; 19 Department of Emergency Medicine, University of Massachusetts Chan Medical School, Worcester, Massachusetts; 20 Department of Emergency Medicine, Vanderbilt University Medical Center, Nashville, TN; 21 Department of Emergency Medicine, Henry Ford Health System, Detroit, Michigan; 22 Department of Emergency Medicine, Indiana University School of Medicine, Indianapolis; 23 Department of Emergency Medicine, University of Florida College of Medicine-Jacksonville; 24 Department of Emergency Medicine, Cooper Medical School of Rowan University, Camden, New Jersey; 25 Department of Emergency Medicine, Ohio State University College of Medicine, Columbus,; 26 College of Nursing, Ohio State University, Columbus; 27 Department of Emergency Medicine, Oakland University William Beaumont School of Medicine, Rochester, Michigan; 28 Department of Surgery, Division of Traumatology, Surgical Critical Care and Emergency Surgery, Perelman School of Medicine, University of Pennsylvania, Philadelphia; 29 Department of Neurosurgery, Perelman School of Medicine, University of Pennsylvania, Philadelphia,; 30 Department of Emergency Medicine, Einstein Healthcare Network, Philadelphia, Pennsylvania; 31 Department of Emergency Medicine, Sidney Kimmel Medical College, Thomas Jefferson University, Philadelphia, Pennsylvania; 32 Department of Emergency Medicine, Wayne State University, Ascension St John Hospital, Detroit, Michigan; 33 Department of Emergency Medicine, Massachusetts General Hospital, Boston; 34 Department of Emergency Medicine, Brigham and Women’s Hospital, Boston, Massachusetts; 35 Department of Emergency Medicine, Trinity Health-Ann Arbor, Ypsilanti, Michigan; 36 Department of Emergency Medicine, University of Massachusetts Medical School-Baystate, Springfield; 37 Department of Emergency Medicine, McGovern Medical School at UTHealth, Houston, Texas; 38 Department of Emergency Medicine, Harvard Medical School, Boston, Massachusetts; 39 Department of Health Care Policy, Harvard Medical School, Boston, Massachusetts; 40 Department of Epidemiology, Harvard T.H. Chan School of Public Health, Harvard University, Boston, Massachusetts; 41 Department of Emergency Medicine, University of North Carolina at Chapel Hill; 42 Institute for Trauma Recovery, Department of Psychiatry, University of North Carolina at Chapel Hill

## Abstract

**Importance:**

Differences in neighborhood socioeconomic characteristics are important considerations in understanding differences in risk vs resilience in mental health. Neighborhood disadvantage is associated with alterations in the function and structure of threat neurocircuitry.

**Objective:**

To investigate associations of neighborhood disadvantage with white and gray matter and neural reactivity to positive and negative stimuli in the context of trauma exposure.

**Design, Setting, and Participants:**

In this cross-sectional study, survivors of trauma who completed sociodemographic and posttraumatic symptom assessments and neuroimaging were recruited as part of the Advancing Understanding of Recovery After Trauma (AURORA) study between September 2017 and June 2021. Data analysis was performed from October 25, 2022, to February 15, 2023.

**Exposure:**

Neighborhood disadvantage was measured with the Area Deprivation Index (ADI) for each participant home address.

**Main Outcomes and Measures:**

Participants completed separate threat and reward tasks during functional magnetic resonance imaging. Diffusion-weighted and high-resolution structural images were also collected. Linear models assessed the association of ADI with reactivity, microstructure, and macrostructure of a priori regions of interest after adjusting for income, lifetime trauma, sex at birth, and age. A moderated-mediation model tested whether ADI was associated with neural activity via microstructural changes and if this was modulated by PTSD symptoms.

**Results:**

A total of 280 participants (183 females [65.4%]; mean [SD] age, 35.39 [13.29] years) completed the threat task and 244 participants (156 females [63.9%]; mean [SD] age, 35.10 [13.26] years) completed the reward task. Higher ADI (per 1-unit increase) was associated with greater insula (*t*_274_ = 3.20; β = 0.20; corrected *P* = .008) and anterior cingulate cortex (ACC; *t*_274_ = 2.56; β = 0.16; corrected *P* = .04) threat-related activity after considering covariates, but ADI was not associated with reward reactivity. Greater disadvantage was also associated with altered microstructure of the cingulum bundle (*t*_274_ = 3.48; β = 0.21; corrected *P* = .001) and gray matter morphology of the ACC (cortical thickness: *t*_273_ = −2.29; β = −0.13; corrected *P* = .02; surface area: *t*_273_ = 2.53; β = 0.13; corrected *P* = .02). The moderated-mediation model revealed that ADI was associated with ACC threat reactivity via cingulum microstructural changes (index of moderated mediation = −0.02). However, this mediation was only present in individuals with greater PTSD symptom severity (at the mean: β = −0.17; standard error = 0.06, *t*= −2.28; *P* = .007; at 1 SD above the mean: β = −0.28; standard error = 0.08; *t* = −3.35; *P* < .001).

**Conclusions and Relevance:**

In this study, neighborhood disadvantage was associated with neurobiology that supports threat processing, revealing associations of neighborhood disadvantage with neural susceptibility for PTSD and suggesting how altered structure-function associations may complicate symptoms. Future work should investigate specific components of neighborhood disadvantage that may be associated with these outcomes.

## Key Points

**Question:**

Is neighborhood disadvantage associated with neural reactivity to threat and reward in the early aftermath of trauma?

**Findings:**

In this cross-sectional study of 280 and 244 survivors of recent trauma completing threat and reward tasks, respectively, greater neighborhood disadvantage was associated with greater neural response to threat but not reward. For individuals with more severe posttraumatic stress symptoms, threat reactivity in the anterior cingulate cortex was partly explained by the association of neighborhood disadvantage with changes in underlying brain structure.

**Meaning:**

This study found that in survivors of trauma, neighborhood disadvantage had specific associations with threat-related neurobiology and that the association with altered neural features may vary based on levels of posttraumatic stress disorder symptoms.

## Teaser

This cross-sectional study investigates associations between neighborhood deprivation and neural reactivity to treat and reward among survivors of recent trauma.

## Introduction

Approximately 24 million individuals live in socioeconomically disadvantaged neighborhoods within the US.^1^ Exposure to neighborhood disadvantage is associated with higher levels of psychosocial stress and increased risk for developing mental health symptoms beyond individual and family factors.^2,3,4^ However, the mechanisms linking neighborhood disadvantage with psychiatric disorders remain unclear.^5^ Several frameworks propose that neighborhood disadvantage is a form of chronic stress that impacts changes in neural systems involved in threat and reward processing.^6,7,8^ Characterizing these neural outcomes may be critical for understanding the association between neighborhood-level chronic stress and mental health.

Neighborhood disadvantage is associated with changes in brain regions (eg, the hippocampus, amygdala, and ventromedial prefrontal cortex) supporting the detection of threat and expression or regulation of threat responses.^6,7,9,10,11^ In young adults, greater disadvantage was inversely associated with quantitative anisotropy within the uncinate fasciculus, cingulum bundle, and fornix and stria terminalis.^12^ Furthermore, individuals living in more disadvantaged neighborhoods showed greater amygdala reactivity to fearful faces compared with neutral faces in adolescence and adulthood.^13,14^ Previous work has also suggested that differences in threat processing regions by race and ethnicity are partly associated with differential exposure to neighborhood disadvantage (reviewed in George et al^15^). In the US, Black and Hispanic individuals are disproportionately exposed to neighborhood disadvantage compared with White individuals, a disparity that was created and is maintained by structural racism.^16^ In survivors of recent trauma, neighborhood disadvantage helped explain differences by race and ethnicity in amygdala resting-state connectivity with regions involved in threat processing (eg, the insula).^17^ Prior work found that Black youths living in more disadvantaged neighborhoods showed greater activity in the anterior cingulate cortex (ACC) in response to social threat compared with White youths.^8^ Together, these studies suggest that neighborhood disadvantage may be associated with changes in multiple regions underlying threat processing.^8,12,13^

Far fewer studies have investigated the potential association of neighborhood disadvantage with reward-related activity. Threat and reward represent opposite ends of the valence spectrum that categorizes the degree to which a stimulus is aversive or desirable.^18^ While threat-related circuitry receives the most attention, reward circuits are unequivocally critical for survival.^18^ The combination of the 2 processes leads to environmentally driven behavioral changes to minimize threat and maximize reward.^18^ The limited work on neighborhood disadvantage and reward processing underscores the pursuit to maximize reward.^8^ Youths living in more disadvantaged neighborhoods displayed greater reactivity to a social reward (ie, a happy face) in canonical reward circuitry, including the nucleus accumbens and putamen, as well as the ACC and amygdala.^8^ Therefore, there is a need to understand whether there is a differential association between neighborhood disadvantage and neurobiology that depends on positive vs negative valence responses.

Limited research to date has investigated if the association of neighborhood disadvantage with neurocircuitry may augment susceptibility to posttraumatic stress disorder (PTSD) in survivors of trauma. PTSD is associated with similar neural alterations observed in individuals with greater neighborhood disadvantage (ie, greater reactivity to threat and altered reactivity to reward).^19,20,21^ In survivors of recent trauma, greater neighborhood disadvantage was associated with reduced hippocampal volume and decreased ventromedial prefrontal cortex thickness, 2 possible risk factors associated with PTSD, even after adjusting for PTSD symptoms.^10^ However, the prior work adjusted for PTSD symptoms and did not investigate whether neighborhood disadvantage was associated with amplified PTSD symptoms via neighborhood disadvantage. Thus, it remains unclear how neurobiological outcomes associated with neighborhood disadvantage may interact with or facilitate neurobiological outcomes associated with trauma exposure.

This study used data from the Advancing Understanding of Recovery After Trauma (AURORA) study to investigate associations of neighborhood disadvantage with neural reactivity to reward and threat, brain structure, and PTSD symptoms. We expected that neighborhood disadvantage would be associated with greater threat and reward reactivity within a priori threat- and reward-related brain regions. We further hypothesized that neighborhood disadvantage would be associated with white matter microstructure of major tracts underlying significant activity of a priori regions of interest. We also tested the hypothesis that activation to threat or reward was mediated by differences in microstructure. Finally, we hypothesized that associations between neighborhood disadvantage and threat-related regions would be stronger for individuals with greater PTSD symptoms. Findings from this investigation may provide insight into neural outcomes associated with neighborhood disadvantage and the interplay between neural structure and function in the acute aftermath of trauma.

## Methods

### Participants

Individuals were recruited from emergency departments within 72 hours of a traumatic injury as part of the AURORA study^22^ between September 2017 and June 2021. Participants underwent neuroimaging at 1 of 5 neuroimaging sites (Atlanta, Georgia; Belmont, Massachusetts; Philadelphia, Pennsylvania; St Louis, Missouri; and Detroit, Michigan) approximately 2 weeks after the trauma.^22,23,24^ Procedures were approved by each site’s institutional review board. Individuals provided written informed consent and were financially compensated for their participation. This study is reported following the Strengthening the Reporting of Observational Studies in Epidemiology (STROBE) reporting guideline. The included neuroimaging data have been previously reported, but these analyses are unique.^17,24,25^

To retain the largest sample size, participants were not required to have completed both tasks (see eFigure 1 in Supplement 1 for study flowchart). Participants were included in the threat and reward sample if they had useable functional magnetic resonance imaging (fMRI) during the respective task, T1-weighted images, and diffusion tensor imaging (DTI) data. Of 344 participants with useable threat fMRI and DTI data, 51 participants were missing self-report data and 13 participants could not be successfully geocoded. Of 307 participants with useable reward fMRI and DTI data, 51 participants were excluded because they were missing self-report data and 12 participants could not be geocoded. Therefore, 280 participants were in the final threat sample and 244 participants were in the final reward sample.

### Demographics and Psychometric Assessment

In the emergency department, participants self-reported their sex at birth (as listed on their birth certificate), age, and race and ethnicity (queried separately). Due to confidentiality concerns arising from small sample sizes within several racial groups, race and ethnicity were merged into a single ethnoracial variable that included 4 groups: Hispanic, non-Hispanic Black, non-Hispanic White, and other (including American Indian, Asian, Pacific Islander, and other). At the 2-week visit, participants reported their annual household income, which was transformed into a semicontinuous variable such that every 1-unit increase corresponded to an additional $20 000 to $25 000 per year. In addition, participants were administered the following assessments (eMethods in the Supplement) at 2-weeks after the trauma (at the time of scanning) and queried about their symptoms over the past 2 weeks. PTSD symptoms were assessed using the PTSD Symptom Checklist for the *Diagnostic and Statistical Manual of Mental Disorders* (Fifth Edition) (*DSM-5* [PCL-5]).^26^ Lifetime trauma was evaluated using the Life Events Checklist for *DSM-5*, and depression symptoms were measured using the Patient-Reported Outcomes Measurement Information System (PROMIS) Depression instrument.^27^

### Neighborhood Disadvantage

Neighborhood disadvantage was assessed using the Area Deprivation Index (ADI) version 3.1 2019 downloaded from the tool website^28,29,30,31^ obtained from geocoding each participant home address (eMethods in Supplement 1). ADI considers factors collected as part of the American Community Survey (US Census) that represent income, education, employment, and housing quality. Using the survey’s 5-year estimates, 17 factors are weighted to create a single variable reflecting the block group’s neighborhood socioeconomic position relative to all other block groups (smallest publicly available geographical unit) in the US. The weighted variable is converted into percentiles, such that a national ADI ranking of 100 indicates a block group that is the most disadvantaged compared with all other block groups in the US.

### MRI Acquisition

Neuroimaging data were collected across 5 sites with harmonized acquisition protocols on Siemens 3T MRI scanners (eTable 1 in Supplement 1). Preprocessing was performed using fMRIPrep version 1.2.2, a Nipype-based pipeline, as reported in previous work (eMethods in Supplement 1).^23,24,32^

### Functional Tasks

Participants completed a threat and reward task (eMethods in Supplement 1) to probe neural activity during negatively and positively valanced tasks.^23,24,32^ In the threat task, participants viewed blocks of faces depicting fearful or neutral expressions (from the Ekman Library).^33^ The reward task was a modified high-low card guessing game.^34^

### MRI Preprocessing and First-Level Models

As part of the fMRIprep pipeline, brain surfaces were reconstructed using recon-all from FreeSurfer version 6.0.1 (Laboratory for Computational Neuroimaging at the Athinoula A. Martinos Center for Biomedical Imaging), which was used to conduct cortical parcellation.^35^ First-level analyses are described in the eMethods in Supplement 1. Contrasts for bilateral anatomically defined region of interest (ROI) extraction included fearful > neutral blocks for the threat task and gains > losses trials for the reward task. ROIs were selected based on previous work^24^ and defined anatomically using the Automated Anatomical Atlas.^24^ ROIs for the threat task included the amygdala, insula, subgenual anterior cingulate cortex, and ACC. Reward ROIs included the nucleus accumbens, orbitofrontal cortex, ACC, insula, and amygdala.

Fractional anisotropy (FA) and mean diffusivity (MD) values were generated from diffusion-weighted images (eMethods in Supplement 1). We considered the cingulum-cingulate gyrus (CGC) and cingulate-hippocampal gyrus (CGH) given prior observations in threat and reward components of PTSD.^23,36^ Given that there were no specific hypotheses regarding laterality, mean FA and MD values were calculated across hemispheres.

### Statistical Analysis

Analyses were completed in R statistical software version 4.1.2 (R Project for Statistical Computing). Pearson correlation tests were conducted to examine the correlation between PCL-5 scores and ADI in both samples. A 1-way analysis of variance (ANOVA) was also performed to evaluate differences in ADI and income between study sites. Ethnoracial group was not included in any analyses given that the variable serves as proxy for racism-related stress and inequitable exposures, including income and neighborhood disadvantage (eFigure 2 and eFigure 3 in Supplement 1).^37,38^

General linear models (GLMs) were used to test whether ADI was associated with task-based activity in ROIs (5 threat related and 5 reward related) and included income, lifetime trauma, sex, and age as covariates. A Holm-Bonferroni correction was applied for each set of models (eg, models examining threat-related ROIs). A corrected α level of .05 was used for all statistical tests, and the corrected *P* value is reported unless otherwise specified. All tests were 2-sided. Finally, to further investigate whether the association of ADI with reactivity was valence specific, we compared standardized regression coefficients between significant ROIs of the threat task and the reward task using a *Z* test (eMethods in Supplement 1).^39^

Microstructure (ie, FA and MD) and macrostructure (post hoc exploratory tests with cortical thickness and surface area) (eTable 3 in Supplement 1) were examined in regions where significant task-related outcomes were observed and based on voxel-wise ROI analysis (eMethods in Supplement 1). GLMs tested the association of ADI with morphology after adjusting for income, lifetime trauma, sex, and age (eMethods and eFigure 4 in Supplement 1). A Holm-Bonferroni correction was applied for each set of models (ie, cortical thickness and surface area of an ROI).

We next analyzed whether brain microstructure mediated the association between ADI and task reactivity (eMethods and eFigure 6 in Supplement 1). Finally, a moderated mediation model (Process macro for R model 14)^40^ was conducted using a bootstrapping approach (10 000 iterations) to assess whether FA values mediated the association between ADI and task-related activity and if the mediation was dependent on PCL-5 scores. To assess whether the moderated mediation was significant, the index of moderated mediation was examined and post hoc simple slope tests were conducted to probe the moderation at different levels of PTSD symptoms (eMethods in Supplement 1). In addition to the primary analysis, we reran the moderated mediation model with PROMIS scores (eMethods and eFigure 7 in Supplement 1). Data analysis was performed from October 25, 2022, to February 15, 2023.

## Results

### Sample Characteristics

A total of 244 participants (156 females [63.9%]; mean [SD] age, 35.10 [13.26] years; 39 Hispanic [16.0%], 105 non-Hispanic Black [43.0%], and 88 non-Hispanic White [36.0%]) completed the reward task and 280 participants (183 females [65.4%]; mean [SD] age, 35.39 [13.29] years; 45 Hispanic [16.1%], 128 non-Hispanic Black (45.7%), and 95 non-Hispanic White [33.9%) completed the threat task; demographics are presented in Table 1. ADI was not correlated with PTSD symptoms in threat (*r*_278_ = 0.02; *P* = .69) or reward (*r*_242_ = 0.06; *P* = .35) samples. As expected, 1-way ANOVAs revealed that ADI and income significantly varied by site (eResults in Supplement 1); therefore, site was not included as a covariate to prevent issues of multicollinearity with our variable of interest.

**Table 1.** Sample Characteristics

Characteristic	Participants, No. (%)	
	Threat (n = 280)	Reward (n = 244)
Sex at birth		
Female	183 (65.4)	156 (63.9)
Male	97 (34.6)	88 (36.1)
Age, mean (SD) [range], y	35.39 (13.29) [18-70]	35.10 (13.26) [18-70]
Ethnoracial group		
Hispanic	45 (16.1)	39 (16.0)
Non-Hispanic Black	128 (45.7)	105 (43.0)
Non-Hispanic White	95 (33.9)	88 (36.0)
Non-Hispanic other^a^	12 (4.3)	12 (5.0)
Income, $		
<19 000	75 (26.8)	70 (28.7)
19 001-35 000	96 (34.3)	78 (32.0)
35 001-50 000	38 (13.6)	32 (13.1)
50 001-75 000	25 (8.9)	24 (9.8)
75 001-100 000	18 (6.4)	15 (6.2)
>100 000	28 (10)	25 (10.2)
Education		
≤High school	93 (33.2)	84 (34.4)
≥Some college	187 (66.8)	160 (65.6)
Participant measure, mean (SD) [range]		
Area Deprivation Index	58.12 (29.76) [4-100]	57.06 (28.57) [5-100]
Depression symptoms (PROMIS score)	54.8 (9.79) [37-81]	54.4 (9.70) [37-81]
Lifetime trauma (LEC-5 score)	9.28 (10.33) [0-63]	8.56 (9.34) [0-43]
PTSD symptoms (PCL-5 score)	29.56 (17.51) [0-79]	29.35 (16.78) [0-79]

Abbreviations: LEC-5, Life Events Checklist for the *Diagnostic and Statistical Manual of Mental Disorders* (Fifth Edition) (*DSM-5*); PCL-5, PTSD Symptom Checklist for the *Diagnostic and Statistical Manual of Mental Disorders* (Fifth Edition) (*DSM-5*); PROMIS, Patient-Reported Outcomes Measurement Information System.

^a^ The non-Hispanic other ethnoracial group included the following responses: American Indian, Asian, Pacific Islander, and other.

### Neighborhood Disadvantage and Neural Reactivity to Threat and Reward

**Figure 1.**
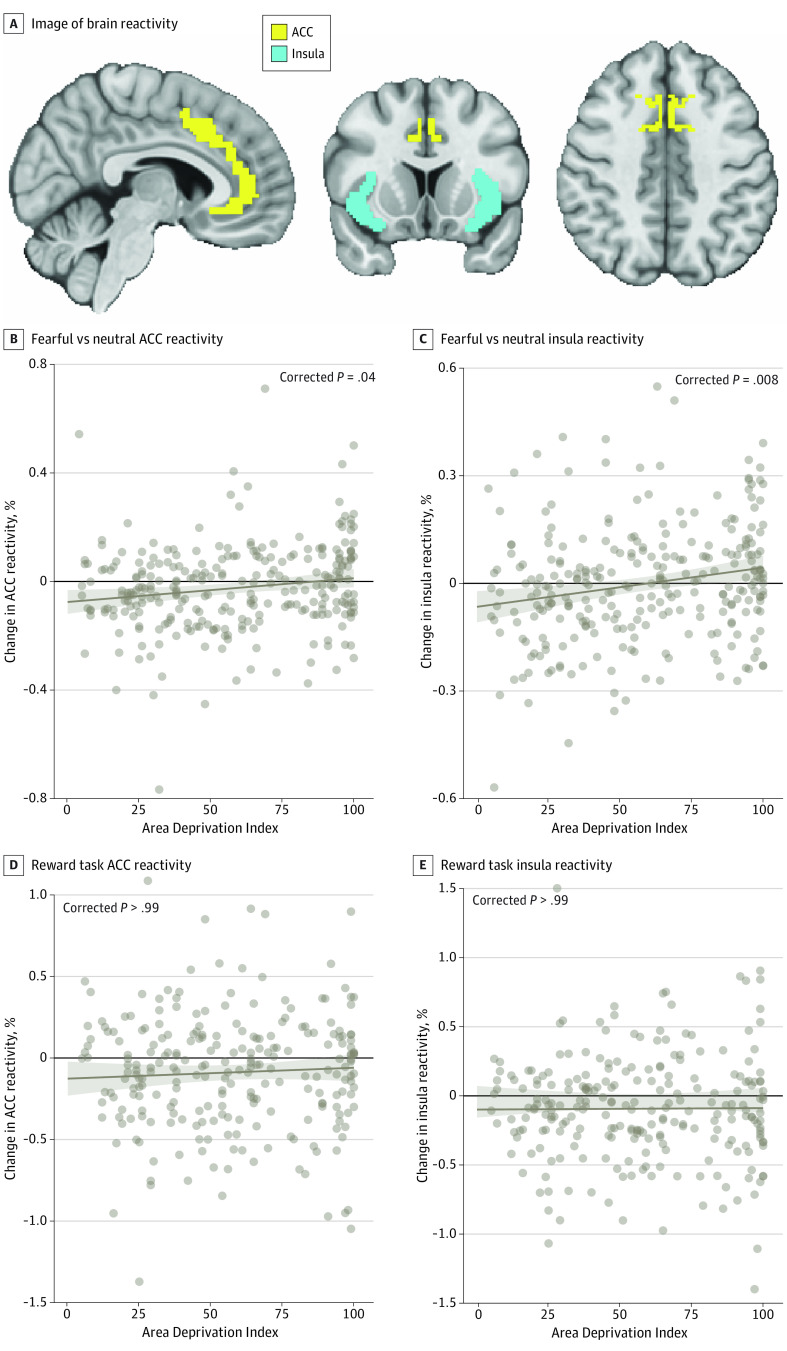
Neighborhood Disadvantage and Reactivity to Threat and Reward A, Neighborhood disadvantage was associated with greater activity to fearful vs neutral faces in the B, anterior cingulate cortex (ACC) and C, insula after covarying for income, lifetime trauma, sex, and age. Higher Area Deprivation Index rankings (per 1-unit increase) were not associated with activity during the reward task in the D, ACC or E, insula. Marginal effects plots depict estimated values (brown regression line) for activity at each Area Deprivation Index ranking. Shaded areas indicate 95% CIs for marginal effects; points, observed data.

In GLMs, higher ADI (per 1-unit increase) was associated with greater threat reactivity within the ACC (*t*_274_ = 2.56; β = 0.16; corrected *P* = .04) (Figure 1A) and insula (*t*_274_ = 3.20; β = 0.20; corrected *P* = .008) (Figure 1B) after covarying for income, lifetime trauma, sex, and age (Table 2). There were no associations between ADI and other ROIs after correction for multiple comparisons. ADI was not associated with differential response to gains vs losses in any reward-related ROI.

**Table 2.** General Linear Models for Task Reactivity

ROI	Variable^a^	Standardized coefficient, β	*t* Statistic	Uncorrected *P* value
**Threat task (n = 280)**				
Hippocampus	Intercept	NA	−1.86	.06
	ADI	0.15	2.30	.02
	Age	0.05	0.87	.39
	Sex	0.01	0.09	.93
	Income	−0.03	−0.49	.63
	LEC-5	0.04	0.61	.54
Amygdala	Intercept	NA	−1.57	.12
	ADI	0.09	1.50	.14
	Age	0.09	1.38	.17
	Sex	0.07	1.22	.23
	Income	<−0.01	−0.04	.97
	LEC-5	0.11	1.87	.06
ACC	Intercept	NA	−1.59	.11
	ADI	0.16	2.56	.01
	Age	<−0.01	−0.05	.96
	Sex	−0.01	−0.24	.81
	Income	<0.01	<.01	>.99
	LEC-5	−0.02	−0.35	.73
Subgenual ACC	Intercept	NA	−0.77	.44
	ADI	0.02	0.26	.80
	Age	0.04	0.67	.50
	Sex	−0.03	−0.49	.63
	Income	0.05	0.73	.47
	LEC-5	−0.06	−0.91	.37
Insula	Intercept	NA	−1.16	.25
	ADI	0.20	3.20	.001
	Age	−0.02	−0.38	.70
	Sex	−0.04	−0.73	.47
	Income	0.01	0.17	.87
	LEC-5	<0.01	0.05	.96
**Reward task (n = 244)**				
Amygdala	Intercept	NA	2.76	.006
	ADI	−0.07	−0.91	.37
	Age	−0.10	−1.34	.18
	Sex	<0.01	0.07	.94
	Income	0.07	0.97	.33
	LEC-5	−0.13	−2.04	.04
Nucleus accumbens	Intercept	NA	2.53	.012
	ADI	0.10	1.41	.16
	Age	−0.04	−0.66	.51
	Sex	−0.04	−0.62	.54
	Income	0.08	1.17	.24
	LEC-5	0.03	0.53	.60
OFC	Intercept	NA	−0.80	.43
	ADI	<0.01	0.15	.89
	Age	0.07	1.06	.29
	Sex	−0.02	−0.33	.74
	Income	0.07	1.03	.31
	LEC-5	−0.05	−0.75	.46
ACC	Intercept	NA	−2.32	.02
	ADI	0.05	0.80	.42
	Age	0.05	0.79	.43
	Sex	−0.10	−1.52	.13
	Income	0.20	3.05	.002
	LEC-5	−0.05	−0.76	.45
Insula	Intercept	NA	−1.33	.18
	ADI	0.01	0.12	.91
	Age	<−0.01	−0.02	.99
	Sex	−0.03	−0.43	.67
	Income	0.13	1.88	.06
	LEC-5	−0.06	−0.85	.40

Abbreviations: ACC, anterior cingulate cortex; ADI, Area Deprivation Index; OFC, orbitofrontal cortex; LEC-5, Life Events Checklist for the *Diagnostic and Statistical Manual of Mental Disorders* (Fifth Edition) (*DSM-5*); NA, not applicable; ROI, region of interest.

^a^ All variables except sex were continuous and defined as per-unit increases.

### Neighborhood Disadvantage and ACC Microstructure

**Figure 2.**
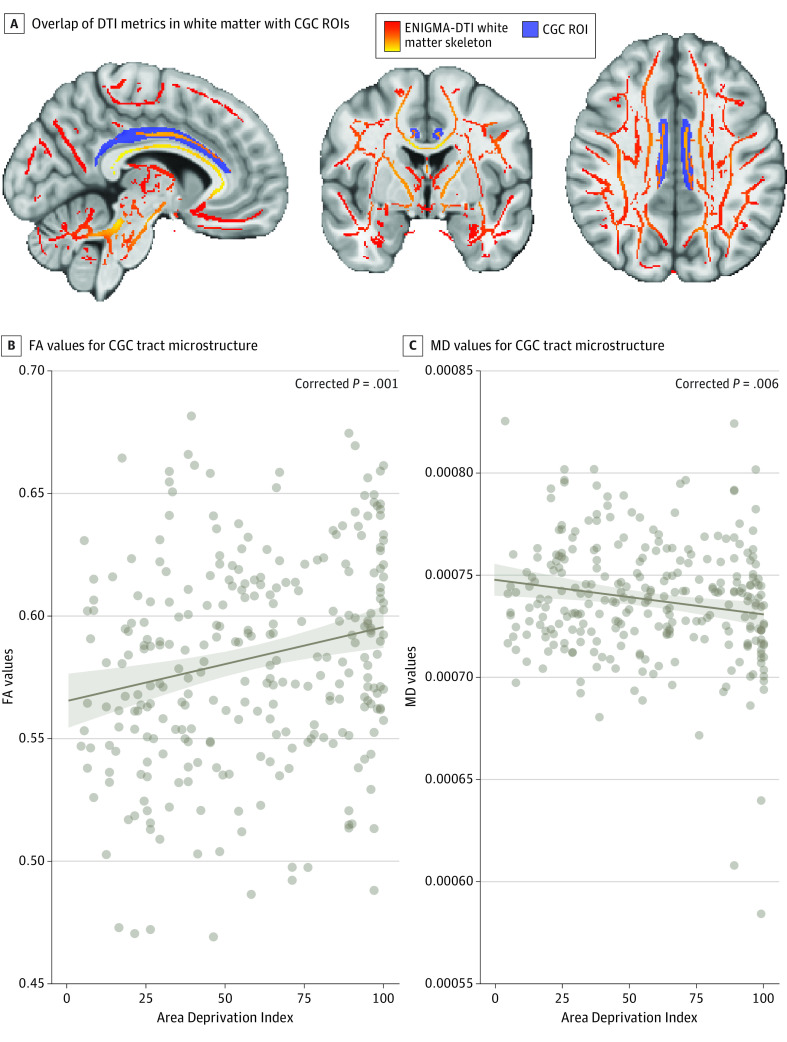
Neighborhood Disadvantage and Brain Microstructure Neighborhood disadvantage was associated with cingulum-cingulate gyrus (CGC) microstructure. A, Brain figures depict the overlap of the Enhancing Neuroimaging Genetics Through Meta-Analysis (ENIGMA) consortium–diffusion tensor imaging (DTI) white matter skeleton and CGC region of interest (ROI). Higher Area Deprivation Index (per 1-unit increase) was associated with higher B, fractional anisotropy (FA) and C, lower mean diffusivity (MD) values after covarying for income, lifetime trauma, sex, and age. Marginal effects plots depict estimated values (brown regression line) for CGC at each Area Deprivation Index ranking. Shaded areas indicate 95% CIs for marginal effects; points, observed data.

Given that neighborhood disadvantage was associated with threat-related reactivity, we further examined whether ADI was associated with DTI metrics in white matter tracts (eg, CGC and CGH) involved in threat processing, which are also associated with PTSD (eTable 2 in Supplement 1). In the threat sample, higher ADI (per 1-unit increase) was associated with higher FA (*t*_274_ = 3.48; β = 0.21; corrected *P* = .001) and lower MD (*t*_274_ = −2.79; β = −0.17; corrected *P* = .006) values in the CGC after adjusting for income, lifetime trauma, sex, and age (Figure 2). In addition, higher ADI was associated with gray matter morphology of the ACC, including reduced cortical thickness (*t*_273_ = −2.29; β = −0.13; corrected *P* = .02) and greater surface area (*t*_273_ = 2.53; β = 0.13; corrected *P* = .02) after covarying for age, sex, income, total intracranial volume, and lifetime trauma (eResults in Supplement 1). ADI was not associated with FA or MD values in the CGH.

### PTSD Symptoms and the Association Between Microstructure and Threat Reactivity

**Figure 3.**
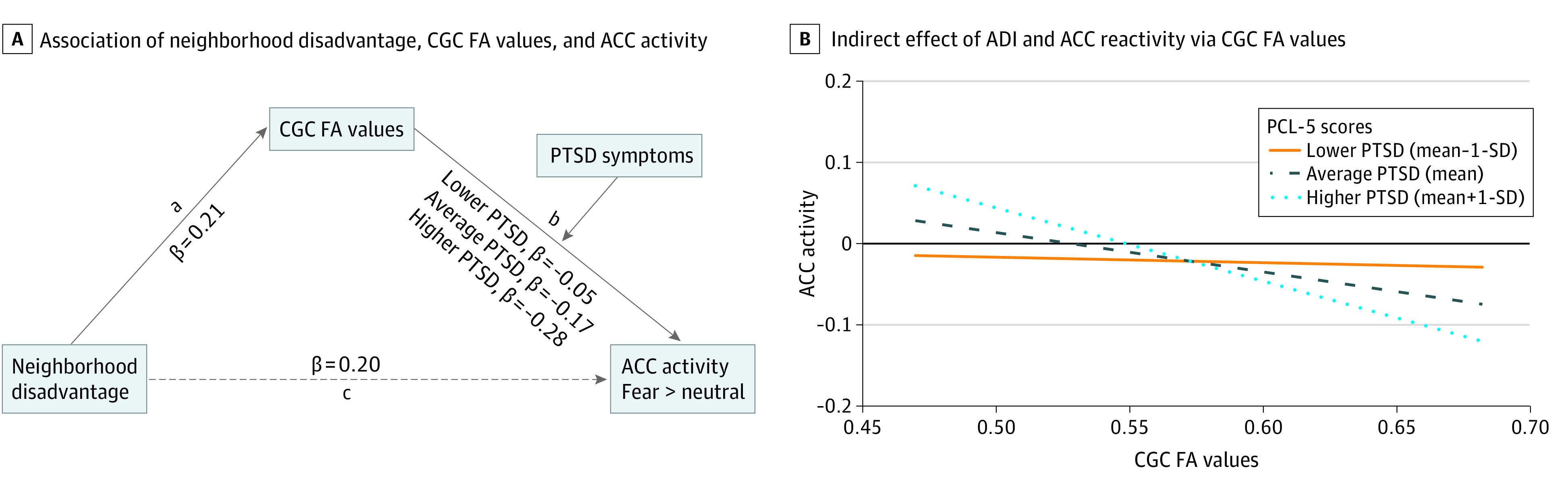
Associations Between Neighborhood Disadvantage, Microstructure, Threat Reactivity, and Posttraumatic Stress Disorder (PTSD) Symptoms A, Neighborhood disadvantage was associated with anterior cingulate cortex (ACC) threat reactivity via cingulum-cingulate gyrus (CGC) fractional anisotropy (FA) values (path a), and PTSD symptoms moderated the association between microstructure and reactivity (path b), even after adjusting for income, lifetime trauma, sex, and age. B, There were conditional indirect effects of Area Deprivation Index (ADI) and ACC reactivity via CGC FA at higher but not lower PTSD symptoms. Coefficients are standardized.

In the threat sample, a moderated mediation model revealed that the association between ADI and ACC reactivity to threat was mediated by PTSD symptoms (Figure 3A). After adjusting for income, lifetime trauma, sex, and age, the *a* path from ADI to CGC FA was significant (β = 0.21; *B*standard error = 0.06; *t* = 3.48; *P* < .001). Greater PTSD symptoms (PCL-5 scores at the mean and 1 SD above the mean) moderated the association between CGC FA and ACC reactivity to threat (interaction β = −0.11; *B*standard error = 0.06; *t* = −2.00; *P* = .046). The overall moderated mediation was significant (index of moderated mediation = −0.02; bootstrapped 95% CI, −0.05 to −0.001).

The conditional indirect effect was strongest in participants who had higher PTSD symptoms (β = −0.17; standard error = 0.06; *t* = −2.28; *P* = .007; 1 SD above the mean: β = −0.28; standard error = 0.08; *t* = −3.35; *P* < .001) but was not present in those with less severe PTSD symptoms (1 SD below the mean) (Figure 3B). Thus, there was an association between CGC microstructure and ACC reactivity to threat only in individuals with greater PTSD symptoms.

## Discussion

Neighborhood disadvantage is a chronic stressor that may interact with trauma in associations with how the brain processes stimuli. In this cross-sectional study, associations of neighborhood disadvantage were valence specific such that greater disadvantage was associated with greater reactivity to threat within the ACC and insula. In turn, ACC reactivity was mediated by underlying microstructure in individuals with higher PTSD symptoms. Surprisingly, neighborhood disadvantage was not associated with reward reactivity. Taken together, the findings suggest that heightened PTSD symptoms may have an interaction, in part, with a unique association between disadvantaged neighborhoods and threat-related structure-function associations of the ACC.

Preclinical and human studies indicate that the ACC is necessary for evaluating stimuli, particularly threatening cues that are not imminent (such as fearful faces),^41^ and then initiating affective responses.^42^ Interestingly, the outcome associated with neighborhood disadvantage was detected in the caudal ACC, a subregion proposed to subserve orientation and expression to threat.^43^ The insula plays a complementary role in initiating affective responses to threat by serving as an interface between appraisal of threat stimuli and internal arousal.^44,45^ Compared with individuals living in more advantaged neighborhoods, individuals from disadvantaged communities encounter more threats to their safety.^8^ More frequent exposure to threat appears to be associated with augmented neural processing of threatening stimuli to salience network nodes,^46^ which may be associated with neural susceptibility for future PTSD. Indeed, a 2021 study from AURORA^24^ found that participants with insula and ACC hyperactivity were more likely to have more severe PTSD symptoms at 3 months after injury. As expected, there were significant ethnoracial differences in neighborhood disadvantage. Future work should continue to investigate how structural inequities are associated with threat processing and consider interactions between other racism-related stressors (eg, racial discrimination), which have been shown to be associated with changes in the insula and ACC.^47,48^ Together, our findings suggest that exposure to neighborhood disadvantage may be associated with increased neural readiness to respond to threat via modification of ACC and insula reactivity.

Given similar associations of PTSD and neighborhood disadvantage with threat reactivity,^49^ we initially expected that amygdala reactivity would be modulated by participant neighborhood disadvantage rankings. However, trauma exposure may have been associated with potentiated amygdala reactivity and obscured outcomes associated with neighborhood disadvantage. AURORA analyses in 2023 did not observe between-group differences in reactivity during the fearful faces task among survivors of recent trauma when stratified by prior sexual trauma^50^ or by ethnoracial group with differing levels of neighborhood disadvantage.^17^ However, differences in amygdala intrinsic connectivity by ethnoracial group were mediated by differential exposure to neighborhood disadvantage.^17^ Earlier work demonstrating associations between socioeconomic measures, including neighborhood disadvantage, and amygdala reactivity was conducted in individuals who had not experienced trauma.^13,14,51^ Thus, conflicting findings suggest that there may be a limit of amygdala threat reactivity in the recent aftermath of trauma.

Neighborhood disadvantage was associated with greater CGC FA values and lower MD values. Intact membrane integrity characterized by lower MD and greater axonal density and coherence reflected by higher FA values are together indicative of greater microstructural integrity.^52^ Greater integrity in this tract may be associated with facilitated communication between the prefrontal cortex and ACC.^53^ Macrostructural features often mirror microstructural changes,^54^ and we found a pattern of reduced cortical thickness and greater surface area. This pattern is present in healthy adults, in line with the theory that neuronal reshaping processes favor surface area expansion rather than increasing cortical thickness.^55^ Thus, individuals from more disadvantaged neighborhoods appeared to exhibit more mature ACC and insula macrostructure.

The association between microstructure and threat reactivity was not the same for all individuals. Only individuals who had mean or greater PTSD symptoms showed a negative association between CGC microstructure and ACC reactivity. Ultimately, outcomes associated with PTSD and neighborhood disadvantage appeared to be associated with similar patterns of activity (ie, heightened ACC reactivity). However, PTSD was associated with reductions in CGC integrity, whereas neighborhood disadvantage was not. Prior work^56^ has suggested that lesser integrity of the CGC was associated with greater psychophysiological responses to threatening cues and lesser integrity is prospectively associated with future PTSD symptoms.^57^ Future directions may include evaluating the association between structure and function in populations with chronic PTSD symptoms to investigate whether this outcome is timing specific.

### Limitations

Several limitations should be considered when interpreting our results. First, our data were cross-sectional and did not offer a temporal ordering of ADI, brain structure, or function. While we relied on previous work suggesting that neighborhood disadvantage can lead to structural alterations,^9,12^ our findings cannot establish a causal relationship. In addition, we derived ADI rankings based off participant home addresses at study enrollment. Unfortunately, we did not capture residential stability (ie, length of residency) or collect previous addresses, which may be critical when considering the dose-dependent association of neighborhood disadvantage exposure. Neighborhood disadvantage was measured with a composite score designed to capture multiple factors. Composite scores are useful insofar as they can reflect the multidimensionality and complexity of neighborhood features; however, they do not offer information on specific factors associated with these outcomes.

There was no association of ADI with reward-related activity. Notably, trauma exposure has been found to be associated with blunted reward reactivity,^19^ whereas neighborhood disadvantage has been associated with greater reward reactivity.^8,58^ Therefore, recent trauma exposure may have been associated with attenuation of reward reactivity and obscured any associations with neighborhood disadvantage, a hypothesis that should be tested in future work with control groups without trauma experience. Another possible explanation may lie in the type of neuroimaging task used; the reward task was a monetary incentive task, whereas the threat task used face stimuli. Future directions include exploring associations of neighborhood disadvantage with outcomes in a single task, which may facilitate a direct comparison of valence. Social reward paradigms, which evoke different patterns of neural activity compared with monetary incentives, may be better situated to explore neural outcomes associated with ADI. Given that neighborhoods exist within social contexts, neural outcomes associated with neighborhood factors may be more evident when underlying social processes are also engaged.

## Conclusions

In this cross-sectional study, neighborhood disadvantage was associated with neurobiology that supports threat processing, and this outcome was more pronounced in individuals with greater PTSD symptoms. These results have implications for neuroscientific studies of trauma and clinical interventions. Regarding neuroscientific studies, elucidating how trauma may be associated with disruptions in structure-function relationships may help to identify novel markers associated with PTSD. Indeed, evaluating the neighborhood context may help to capture important neural variability. Our results suggest that clinical interventions may be better tailored to patients if these interventions consider where patients reside and specific neighborhood characteristics that may be associated with symptom presentation and treatment response. This study adds to the substantial evidence that the association between socioeconomic inequities and mental health may be brain-mediated and emphasizes the pressing need for structural change.

## Notes

Supplement 1.
eMethods.

eResults.
**eTable 1.** Harmonized Magnetic Resonance Imaging Sequences Across Study Sites **eTable 2.** General Linear Models for White Matter Tracts Among 280 Participants **eTable 3.** General Linear Models for Macrostructure Among 280 Participants **eFigure 1.** Flowchart of Study Participants Who Met Inclusion Criteria for Threat or Reward Analyses **eFigure 2.** Neighborhood Disadvantage and Income by Race and Ethnicity **eFigure 3.** Neighborhood Disadvantage and Income by Study Site **eFigure 4.** Anterior Cingulate Cortex and Neighborhood Disadvantage Region of Interest Analysis **eFigure 5.** Neighborhood Disadvantage and Macrostructure **eFigure 6.** Associations Between Neighborhood Disadvantage, Microstructure, and Threat Reactivity **eFigure 7.** Associations Between Neighborhood Disadvantage, Microstructure, Threat Reactivity, and Depression Symptoms
eReferences.

Supplement 2.
Data Sharing Statement
