# Supplementary material for: Neighborhood Disadvantage and Neural Correlates of Threat and Reward Processing in Survivors of Recent Trauma
Source: JAMA Netw Open. 2023 Sep 18;6(9):e2334483. doi: 10.1001/jamanetworkopen.2023.34483 (PMC10507487; doi:10.1001/jamanetworkopen.2023.34483)
Supplement: Supplement 1. — eMethods. eResults. eTable 1. Harmonized Magnetic Resonance Imaging Sequences Across Study Sites eTable 2. General Linear Models for White Matter Tracts Among 280 Participants eTable 3. General Linear Models for Macrostructure Among 280 Participants eFigure 1. Flowchart of Study Participants Who Met Inclusion Criteria for Threat or Reward Analyses eFigure 2. Neighborhood Disadvantage and Income by Race and Ethnicity eFigure 3. Neighborhood Disadvantage and Income by Study Site eFigure 4. Anterior Cingulate Cortex and Neighborhood Disadvantage Region of Interest Analysis eFigure 5. Neighborhood Disadvantage and Macrostructure eFigure 6. Associations Between Neighborhood Disadvantage, Microstructure, and Threat Reactivity eFigure 7. Associations Between Neighborhood Disadvantage, Microstructure, Threat Reactivity, and Depression Symptoms eReferences. [file jamanetwopen-e2334483-s001.pdf]

Supplemental Online Content

Webb EK, Ely TD, Rowland GE, et al. Neighborhood disadvantage and neural correlates of threat and reward processing in survivors of recent trauma. *JAMA Netw Open*. 2023;6(9):e2334483. doi:10.1001/jamanetworkopen.2023.34483

eMethods.

eResults.

eTable 1. Harmonized Magnetic Resonance Imaging Sequences Across Study Sites

eTable 2. General Linear Models for White Matter Tracts Among 280 Participants

eTable 3. General Linear Models for Macrostructure Among 280 Participants

eFigure 1. Flowchart of Study Participants Who Met Inclusion Criteria for Threat or Reward Analyses

eFigure 2. Neighborhood Disadvantage and Income by Race and Ethnicity

eFigure 3. Neighborhood Disadvantage and Income by Study Site

eFigure 4. Anterior Cingulate Cortex and Neighborhood Disadvantage Region of Interest Analysis

eFigure 5. Neighborhood Disadvantage and Macrostructure

eFigure 6. Associations Between Neighborhood Disadvantage, Microstructure, and Threat Reactivity

eFigure 7. Associations Between Neighborhood Disadvantage, Microstructure, Threat Reactivity, and Depression Symptoms

eReferences.

This supplemental material has been provided by the authors to give readers additional information about their work.

© 2023 Webb EK et al. *JAMA Network Open.*

eMethods

Study Inclusion/Exclusion Criteria

Individuals were eligible for the study if they were between 18-75 years old and able to read and write in English. Qualifying traumatic events included motor vehicle collisions, physical or sexual assaults, or falls greater than 10 feet, or experiences that otherwise met Diagnostic and Statistical Manual 5th edition criterion A for PTSD [1]. Participants were excluded if they: sustained a solid organ injury greater than grade 1 or had a significant hemorrhage, were intubated, required general anesthesia, or were likely to be admitted for more than three days. Additional exclusion criteria for neuroimaging included the presence of metal or ferromagnetic material in the body, claustrophobia, history of neurodegenerative disorders, or a history of seizures.

Geocoding

Prior to deriving Area Deprivation Index (ADI), participants' addresses at the time of study enrollment were entered into SAS 9. 4 PROC GEOCODE which uses SAS Maps' prebuilt U.S. Street lookup data created with TIGER2Geocode (version 15) to perform location lookup. Addresses that were unable to be matched were visually inspected and a manual look-up was attempted. Approximately 98% of addresses were successfully matched. Each participant's census block group was then matched to the corresponding census block group ADI.

Psychometric Assessment

PTSD symptoms were assessed using the PTSD Symptom Checklist for DSM-5 (PCL-5)[2]. The PCL-5 is a 20 item self-report questionnaire that assesses the presence and severity of various posttraumatic stress symptoms[2]. Participants rated symptoms on a scale of 0 (*not at all*) to 4 (*extremely*) for the severity of each symptom. Depression symptoms were assessed using the Patient-Reported Outcomes Measurement Information System (PROMIS) Depression instrument[3]. The PROMIS questionnaire (short form 8b) has eight-items evaluating depressive symptom frequency scored from 1 (*never)* to 5 (*always*). A raw total score was computed from summing the individual items and then converted to a T-score. The PCL-5 and the PROMIS were administered at 2-weeks post-trauma (at the

© 2023 Webb EK et al. *JAMA Network Open.*

time of scanning), and participants were asked about their symptoms over the past 2 weeks (i.e., since the trauma). In addition, at the 2-week visit, participants completed the Lifetime Events Checklist for DSM-5 (LEC-5) which evaluated whether the participant had experienced, witnessed, or learned about 16 different stressful or traumatic events (e.g., "Serious accident at work, home, or during recreational activity"). A total score was created by summing all responses.

Functional Tasks

In the threat task, participants viewed blocks of faces depicting fearful or neutral expressions (from the Ekman library). Each of the blocks (15 neutral; 15 fearful) were presented for 8s. Block order was counterbalanced across participants. Within a block, 8 different faces were presented for 500ms with an intertrial interval of 500ms. After every 10 blocks, participants were given a rest period of 10s and were instructed to relax with their eyes open. The reward task was a modified high/low card guessing game [4]. Participants viewed cards with a question mark and had 2s to guess whether the card's value was higher or lower than $5. Following a short, jittered delay (2-4s), the card's value and monetary outcome was displayed. Prior to the task, participants were informed they would win $1 for each correct guess and lose $0.50 for each incorrect guess. A total of 40 cards were presented, with a predetermined 20 gains and 20 losses (participants always won $10).

MRI Preprocessing in fMRIPrep

Results included in this manuscript come from preprocessing performed using fMRIPrep v1.2.2 [5,6]. Each T1w (T1-weighted) volume was corrected for INU (intensity non-uniformity) using N4BiasFieldCorrection v2.1.0 [7]and skull-stripped using antsBrainExtraction.sh v2.1.0 (using the OASIS template). Brain surfaces were reconstructed using recon-all from FreeSurfer v6.0.1, and the brain mask estimated previously was refined with a custom variation of the method to reconcile ANTs-derived and FreeSurfer-derived segmentations of the cortical gray-matter of Mindboggle [8].

Spatial normalization to the ICBM 152 Nonlinear Asymmetrical template version 2009c [9] was performed through nonlinear registration with the antsRegistration tool of ANTs v2.1.0 [10], using brain-extracted versions of both T1w volume and template. Brain tissue segmentation of cerebrospinal fluid

© 2023 Webb EK et al. *JAMA Network Open.*

(CSF), white-matter (WM) and gray-matter (GM) was performed on the brain-extracted T1w using fast (FSL v5.0.9). Functional data was slice-time corrected using 3dTshift from AFNI v16.2.07 and motion corrected using mcflirt (FSL v5.0.9). This was followed by co-registration to the corresponding T1w using boundary-based registration with six degrees of freedom, using bbregister (FreeSurfer v6.0.1). Motion correcting transformations, BOLD-to-T1w transformation and T1w-to-template (MNI) warp were concatenated and applied in a single step using antsApplyTransforms (ANTs v2.1.0) using Lanczos interpolation.

Frame-wise displacement was calculated for each functional run using the implementation of Nipype. ICA-based Automatic Removal Of Motion Artifacts (AROMA) was used to generate aggressive noise regressors as well as to create a variant of data that is non-aggressively denoised [11]. For more details of the pipeline see https://fmriprep.readthedocs.io/en/stable/workflows.html. An overall motion threshold was also implemented such that any participant's task data with >15% volumes and ≥1-mm framewise displacement were excluded.

In first-level analyses, as the threat task was a block design, blocks were modeled using separate boxcar functions whereas the event-related reward task gain and loss trials were modeled as separate events convolved with a canonical hemodynamic response function. Contrasts for region of interest extraction (ROI) included *fearful* > *neutral* blocks for the threat task and *gains* > *losses* trials for the reward task. ROIs were selected based on previous work[12] and defined anatomically using the Automated Anatomical Atlas[12]. ROIs for the threat task included the amygdala, insula, Brodmann's area (BA) 25 (corresponds to the subgenual anterior cingulate cortex) and BA32 (corresponds to the ACC). Reward ROIs included the nucleus accumbens (NAcc), OFC, BA32, insula, and amygdala. *A-priori* selection of these ROIs was not based on the participant's showing significant task-related activation. The mean across all voxels in each ROI was extracted from first-level contrasts. For ROIs with hemisphere differentiation, the activity was averaged across left and right seeds.

As described in prior work[13], diffusion weighted images (DWI) were preprocessed using guidelines from the ENIGMA consortium to derive measures of structural connectivity

© 2023 Webb EK et al. *JAMA Network Open.*

(http://enigma.ini.usc.edu/protocols/dti-protocols/). Briefly, DWI data were first corrected for susceptibility using nonlinear warping to the participant's T1w image. Motion and eddy effects were reduced using FSL's eddy function. Finally, DWI data were fit with a tensor model prior to Tract-Based Spatial Statistics processing.

MRI Quality Control and Exclusions

For T1w images, we assessed sitewise differences in MRI quality control metric in MRI-QC [14]: coefficient of joint variation (CJV) of gray and white matter, median intensity nonuniformity (INU), and the signal-to-noise ratio (SNR). CJV is thought to reflect the presence of heavy head motion and artifacts where lower scores reflect better quality data. INU reflects non-anatomically variation in signal intensity across the volume. SNR is the signal to noise ratio of the entire T1-volume across tissue types.

For DWI data, we assessed sitewise differences in MRI quality control metrics derived from initial processing: temporal signal-to-noise ratio (TSNR), maximum outlier voxel intensity (OUTMAX), mean absolute motion (MEANABS), and maximum absolute motion (MAXABS). TSNR is the temporally averaged signal to noise ratio for each dataset.

For functional data, we assessed sitewise differences in MRI quality control metrics in MRI-QC: the AFNI Quality Index (AQI), FD, DVARs, and temporal-signal-to-noise ratio (TSNR). AQI is a general and crude screening tool for motion or scanner artifacts in 4D datasets. AQI is calculated as an average of 1 minus the Spearman rank correlation coefficient for every volume to the median volume in the dataset. Framewise displacement is an estimation of head-movement across the dataset. DVARs is calculated as the derivative of the root-mean-square variance over dataset voxels [15].

Table S1 describes the harmonized MRI acquisition parameters across each site.

© 2023 Webb EK et al. *JAMA Network Open.*

eResults

Assessment of Missing Data and Lost to Follow-up Participants

Figure S1 provides an overview of reasons participants were excluded from the reward and threat samples. A total of 234 participants had *both* reward and threat task neuroimaging data. An additional 10 completed only reward data and 46 completed only threat data. Independent *t*-tests revealed there were no significant differences between the threat sample and the reward sample in regard to ADI ($t(522) = 0.42$, $p = .68$), income ($t(522) = 0.05$, $p = .95$), PCL-5 scores ($t(522) = 0.81$, $p = .42$), age ($t(522) = 0.25$, $p = .80$), or lifetime trauma ($t(522) = 0.83$, $p = .41$).

Ethnoracial Differences in Area Deprivation Index, Lifetime Trauma, and Income

In both samples (threat and reward), one-way ANOVAs with Tukey post-hoc tests (Holm-Bonferroni corrected) revealed Black participants disproportionately resided in disadvantaged neighborhoods (threat sample: ($F(3,275) = 31.72$, $p < .001$; reward sample: $F(3,238) = 27.00$, $p < .001$) and had lower income (threat sample: ($F(3,275) = 9.18$, $p < .001$; reward sample: $F(3,238) = 8.56$, $p < .001$; Tukey's *post-hoc* test results depicted in Figure S2). In line with previous reports in the AURORA sample [16], White participants reported significantly higher lifetime trauma compared to Black participants (threat sample: ($F(3,275) = 2.52$, $p = .06$; reward sample: $F(3,238) = 2.73$, $p = .04$; Tukey's *post-hoc* test $p = .026$). There were no significant ethnoracial differences in PTSD symptoms (threat sample: ($F(3,275) = 2.05$, $p = .11$; reward sample: $F(3,238) = 0.71$, $p = .55$).

Site Differences in Area Deprivation Index and Income

In both samples, one-way ANOVAs revealed ADI significantly differed by site (threat sample: ($F(4,275) = 42.83$, $p < .001$; reward sample: $F(4,239) = 32.7$, $p < .001$). One-way ANOVA revealed significant site differences for income in the threat sample, but not the reward sample (threat sample: ($F(4,275) = 2.46$, $p = .05$; reward sample: $F(4,239) = 1.46$, $p = .22$) significantly differed by site (Figure S3).

Threat vs Reward *β* Comparison

© 2023 Webb EK et al. *JAMA Network Open.*

To further establish whether the effect of ADI was valence specific, we compared the standardized regression coefficients between the significant ROIs of the threat task to the reward task. Comparisons of the β-values using z-tests confirmed that the effect of ADI on threat-related ACC and insula reactivity was significantly different compared to the association between ADI and reward-related ACC and insula reactivity, respectively (ACC: z-value = 2.34, $p = .02$; insula: z-value = 2.26, $p = .02$).

DWI

Following the significant effects of ADI on ACC and insula activity to *fearful* > *neutral* faces, we selected (post-hoc) white matter tracts which are related to activity in these regions: the cingulum-cingulate gyrus (CGC) and the cingulate-hippocampal gyrus (CGH). The CGC and CGH were selected because of their relevance to the ACC activity. The CGC is the upper segment of the cingulum running adjacent to ACC whereas the CGH is the lower segment and connect the cingulum to the hippocampus (Mori et al., 2008).

Our primary aim was to test whether ADI was associated with fractional anisotropy (FA) values in these tracts. However, to evaluate robustness of the effect of ADI on a different feature of white matter microstructure, we also tested whether ADI was associated with mean diffusivity. Results from the GLMs conducted to evaluate the effect of ADI on the tracts are presented in Table S2. Higher ADI rankings were associated with greater CGC FA values and lesser CGC MD values. These findings suggest the effect of ADI is tract-specific and impacts multiple features of microstructure.

Post-Hoc Voxelwise ACC ROI analysis

Following the significant effects of ADI on ACC and insula activity to *fearful* > *neutral* faces, we examined the surface area and volume of these two regions (see main text). As the BA32 seed used for functional task traversed multiple Freesurfer parcellations corresponding to the ACC, we first conducted a supplementary ROI analysis to determine whether the effect of ADI was located in the rostral or caudal ACC. Results of the 3dttest in AFNI revealed a significant effect of ADI after adjusting for age, sex, and income in the caudal section of the ROI (k = 12, X: 4.5, Y: -33.5, Z = 29.5; Figure S4).

Neighborhood disadvantage is associated with ACC and insula macrostructure

© 2023 Webb EK et al. *JAMA Network Open.*

Given our findings of ADI on threat-related function and structure, we completed exploratory *post-hoc* GLMs to examine the effects of ADI on cortical thickness and surface area of regions which showed significant task-related effects. We selected only the caudal ACC and insula for these analyses given the aforementioned findings. The caudal ACC was chosen based on voxelwise analyses of the BA32 seed region (described above) to determine if the original association was most pronounced in either rostral or caudal subregions given the seed traversed both subregions in the Desikan-Killainy atlas.

Results of the GLM models are provided in Table S3. There was a significant relationship between ADI and caudal ACC cortical thickness ($t(273) = -2.29$, $\beta = -0.13$, $p_{corrected} = .02$) (Figure S5). Higher ADI was associated with greater caudal ACC surface area, even after adjusting for age, sex, income, total intracranial volume, and lifetime trauma, ($t(273) = 2.53$, $\beta = 0.13$, $p_{corrected} = .02$). Higher ADI was negatively related to insula cortical thickness ($t(273) = -2.70$, $\beta = -0.15$, $p_{corrected} = .01$) and positively related to surface area ($t(273) = 2.73$, $\beta = 0.13$, $p_{corrected} = .01$) after adjusting for covariates.

Simple Mediation Model

Prior to testing the moderated mediation model presented in the main text, we examined whether CGC FA values mediated the relationship between ADI and ACC reactivity to fearful > neutral faces (Figure S6; $N = 280$). A mediation model (PROCESS model 4) showed there was a significant indirect effect of CCG FA values between neighborhood disadvantage and ACC reactivity ($\beta = -0.035$, SE = 0.017, 95% CI [-0.0722, -0.059]. There was a significant relationship between ADI and CCG FA values (**a** path: $\beta = 0.21$; SE < 0.01, $t = 3.48$, $p < .001$) after adjusting for sex, age, income, and lifetime trauma. There was also a significant relationship between CGC FA values and ACC activity (**b** path: $\beta = -0.17$; SE = 0.24, $t = -2.69$, $p = .008$), after adjusting for ADI, sex, age, income, and lifetime trauma. The direct effect was also significant (**c'** path: $\beta = 0.20$, SE < 0.01 $t = 3.20$, $p = .002$).

This pattern of results revealed an inconsistent mediation. More specifically, neighborhood disadvantage was associated with greater ACC reactivity and higher CGC FA value (positive **a** and **c** paths), however, CGC values were associated with decreased ACC threat reactivity (negative **b** path).

© 2023 Webb EK et al. *JAMA Network Open.*

This pattern suggested another variable (e.g., PTSD symptoms) may be affecting the relationship between CCG FA values and ACC threat reactivity.

PTSD Symptoms Moderate the Relationship Between Microstructure and Threat Reactivity

Planned post-hoc tests of simple slopes (i.e., conditional effects on **b** path) found no statistically significant relationship between CGC FA values and ACC activity in individuals with lower PTSD symptoms (β = -0.05, SE = 0.08, t = -0.64, p = .52). However, there was a statistically significant conditional effect at the average PTSD symptoms (β = -0.17, SE = 0.06, t = -2.28, p = .007) and +1 SD (β = -0.28, SE = 0.08, t = -3.35, p < .001).

Moderated Mediation Analysis with Depression Symptoms

We repeated the moderated mediation analysis (PROCESS macro; model 14; 10,000 bootstrapping iterations) with depression symptoms as the moderator to determine if the observed relationship was specific to PTSD symptoms or related to general distress (Figure S7).

Depression symptoms did not significantly moderate the effect of CGC integrity on ACC reactivity to threat (interaction β = -.07, SE = 0.06, t = -1.13, p = .26). The overall moderated mediation was not significant (index of moderated mediation = -0.02; standard error: 0.02; bootstrapped 95% CI[-0.05, 0.01].

© 2023 Webb EK et al. *JAMA Network Open.*

Table S1. Harmonized MRI Sequences Across Study Sites

	Site 1	Site 2	Site 3	Site 4	Site 5
**Scanner**	Siemens TIM 3T Trio	Siemens TIM 3T Trio	Siemens MAGNETOM 3T Prisma	Siemens 3T Verio	Siemens MAGNETOM 3T Prisma
**Head Coil**	12 Channel	12 Channel	20 Channel	12 Channel	20 Channel
**Modality**					
**T1-weighted**	**TR** = 2530ms, **TEs** = 1.74/3.6/5.46/7.32ms, **TI** = 1260ms, **flip angle** = 7, **FOV** = 256mm, **slices** = 176, **Voxel size** = 1mm x 1mm x 1mm	**TR** = 2530ms, **TEs** = 1.74/3.6/5.46/7.32ms, **TI** = 1260ms, **flip angle** = 7, **FOV** = 256mm, **slices** = 176, **Voxel size** = 1mm x 1mm x 1mm	**TR** = 2300ms, **TE** = 2.96ms, **TI** = 900ms, **flip angle** = 9, **FOV** = 256mm, **slices** = 176, **Voxel size** = 1.2mm x 1.0mm x 12mm	**TR** = 2530ms, **TEs** = 1.74/3.65/5.51/7.72ms, **TI** = 1260ms, **flip angle** = 7, **FOV** = 256mm, **slices** = 176, **Voxel size** = 1mm x 1mm x 1mm	**TR** = 2300ms, **TE** = 2.98ms, **TI** = 900ms, **flip angle** = 9, **FOV** = 256mm, **slices** = 176, **Voxel size** = 1.2mm x 1.0mm x 12mm
**Diffusion Weighted Imaging**	**TR** = 7700ms, **TE** = 85ms, **FOV** = 212mm, **flip angle** = 90, **Volumes** = 71 (64 **b**=1000 s/mm^{2,} 7 b0), **PA-encoded**, **Voxel size** = 2mm x 2mm x 2mm	**TR** = 7700ms, **TE** = 85ms, **FOV** = 212mm, **flip angle** = 90, **Volumes** = 71 (64 **b**=1000 s/mm^{2,} 7 b0), **PA-encoded**, **Voxel size** = 2mm x 2mm x 2mm	**TR** = 7000ms, **TE** = 74ms, **FOV** = 212mm, **flip angle** = 90, **Volumes** = 71 (64 **b**=1000 s/mm^{2,} 7 b0), **PA-encoded**, **Voxel size** = 2mm x 2mm x 2mm	**TR** = 12000ms, **TE** = 85ms, **FOV** = 212mm, **flip angle** = 90, **Volumes** = 71 (64 **b**=1000 s/mm^{2,} 7 b0), **PA-encoded**, **Voxel size** = 2mm x 2mm x 2mm	**TR** = 7700ms, **TE** = 67ms, **FOV** = 212mm, **flip angle** = 90, **Volumes** = 71 (64 **b**=1000 s/mm^{2,} 7 b0), **PA-encoded**, **Voxel size** = 2mm x 2mm x 2mm
**fMRI**	**TR** = 2360ms, **TE** = 30ms, **flip angle** = 70, **FOV** = 212mm, **slices** = 44, **Voxel size** = 3mm x 2.72mm x 2.72mm, 0.5 mm gap	**TR** = 2360ms, **TE** = 30ms, **flip angle** = 70, **FOV** = 212mm, **slices** = 44, **Voxel size** = 3mm x 3mm x 3mm, 0.5 mm gap	**TR** = 2360ms, **TE** = 29ms, **flip angle** = 70, **FOV** = 212mm, **slices** = 44, **Voxel size** = 3mm x 2.72mm x 2.72mm, 0.5 mm gap	**TR** = 2360ms, **TE** = 30ms, **flip angle** = 70, **FOV** = 212mm, **slices** = 42, **Voxel size** = 3mm x 2.72mm x 2.72mm, 0.5 mm gap	**TR** = 2360ms, **TE** = 29ms, **flip angle** = 90, **FOV** = 210mm, **slices** = 44, **Voxel size** = 3mm x 3mm x 2.5mm, 0.5 mm gap

© 2023 Webb EK et al. *JAMA Network Open.*

Table S2. General Linear Models for White Matter Tracts (*N* = 280)

Tract	*Metric*	*Variable*	*Standardized Coefficient*	*t-statistic*	*Uncorrected p-value*
**CGC**	***FA***	Intercept	-	54.34	<.001
		ADI	0.21	3.48	<.001
		Age	-0.22	-3.77	<.001
		Sex	-0.16	-2.88	.004
		Income	0.08	1.32	.19
		LEC-5	0.05	0.85	.40
	MD	Intercept	-	96.57	<.001
		ADI	-0.17	-2.79	.006
		Age	0.03	0.46	.64
		Sex	0.17	2.90	.004
		Income	-0.08	-1.32	.19
		LEC-5	-0.04	-0.67	.50
**CGH**	***FA***	Intercept	-	44.2	<.001
		ADI	-0.03	-0.56	.58
		Age	0.01	0.18	.86
		Sex	-0.20	-3.40	<.001
		Income	0.02	0.32	.75
		LEC-5	0.12	2.03	.04
	MD	Intercept	-	68.18	<.001
		ADI	-0.10	-1.56	.12
		Age	-0.10	-1.73	.08

© 2023 Webb EK et al. *JAMA Network Open.*

		Sex	0.03	0.53	.60
		Income	-0.01	-0.14	.89
		LEC-5	-0.07	-1.13	.26

Abbreviations: **ADI:** Area Deprivation Index; **CGC:** Cingulum (cingulate gyrus section); **CGH:** Cingulum (hippocampal section); **FA:** fractional anisotropy; **LEC-5:** Life Events Checklist; **MD:** mean diffusivity; **ROI:** Region of Interest.

© 2023 Webb EK et al. *JAMA Network Open.*

Table S3. General Linear Models for Macrostructure (*N* = 280)

ROI	*Metric*	*Variable*	*Standardized Coefficient*	*t-statistic*	*Uncorrected p-value*
**Caudal ACC**	***Cortical Thickness***	Intercept	-	24.28	<.001
		ADI	-0.13	-2.29	.02
		Age	-0.34	-6.14	<.001
		Sex	0.14	1.91	.06
		Income	-0.09	-1.68	.09
		ICV	-0.16	-2.14	.03
		LEC-5	-0.06	-1.05	.30
	Surface Area	Intercept	-	-1.26	.21
		ADI	0.13	2.53	.01
		Age	-0.01	-0.21	.83
		Sex	0.17	2.62	<.001
		Income	0.12	2.29	.02
		ICV	0.70	10.48	<.001
		LEC-5	0.09	1.75	.08
**Insula**	***Cortical Thickness***	Intercept	-	31.25	<.001
		ADI	-0.15	-2.70	.007
		Age	-0.48	-8.81	<.001
		Sex	0.09	1.30	<.001
		Income	-0.01	-0.21	.83
		ICV	0.06	0.77	.44
		LEC-5	-0.06	-1.02	.31

© 2023 Webb EK et al. *JAMA Network Open.*

	Surface Area	Intercept	-	4.84	<.001
		ADI	0.13	2.73	.007
		Age	0.02	0.48	.63
		Sex	-0.07	-1.27	<.001
		Income	0.07	1.59	.11
		ICV	0.66	11.23	<.001
		LEC-5	0.05	1.20	.23

Abbreviations: ***ACC:*** Anterior Cingulate Cortex; **ADI:** Area Deprivation Index; **ICV:** Intracranial Volume; **LEC-5:** Life Events Checklist; **ROI:** Region of Interest.

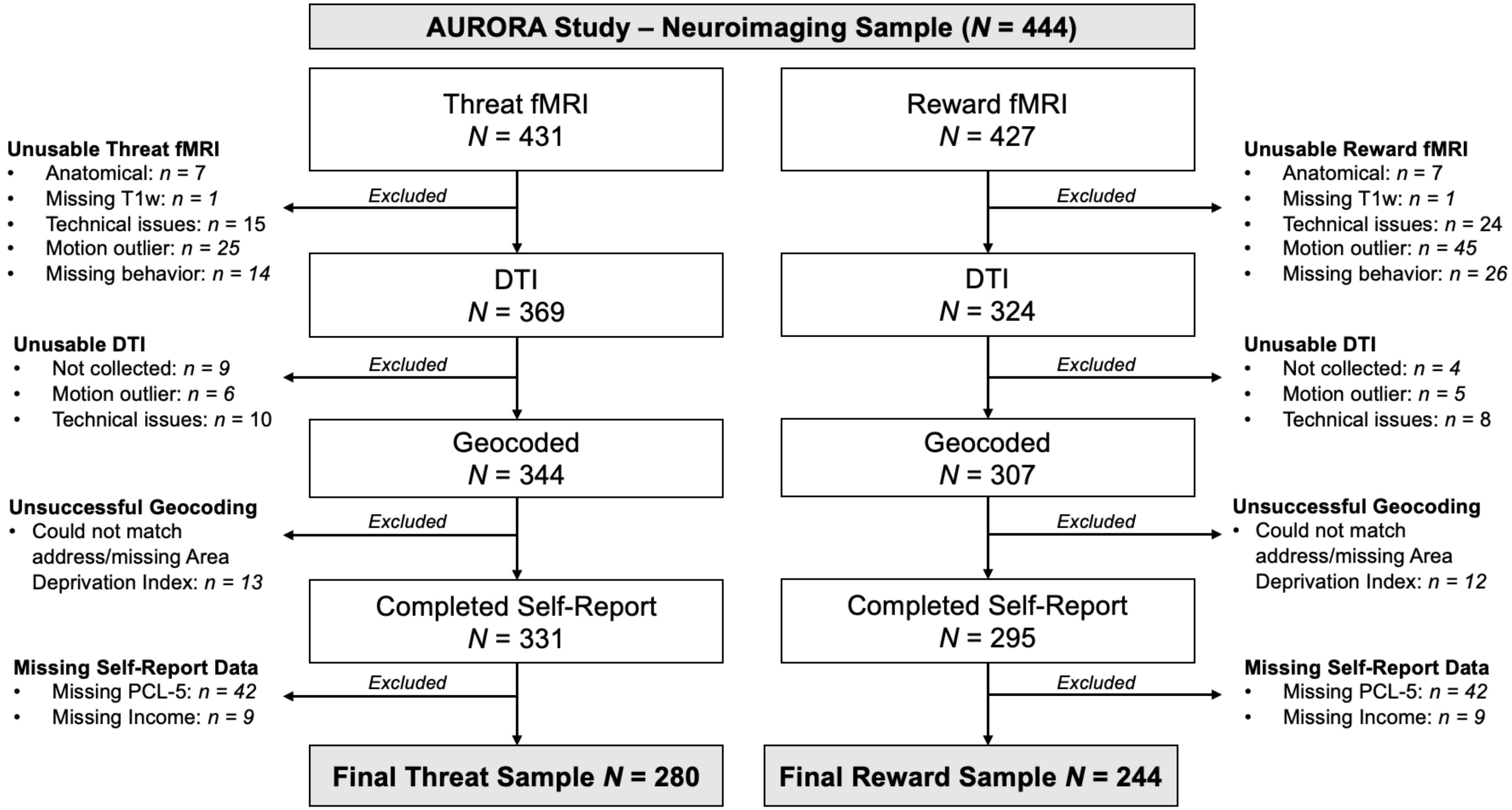

Figure S1. Flowchart of AURORA study participants who met inclusion criteria for the threat or reward analyses.

© 2023 Webb EK et al. *JAMA Network Open.*

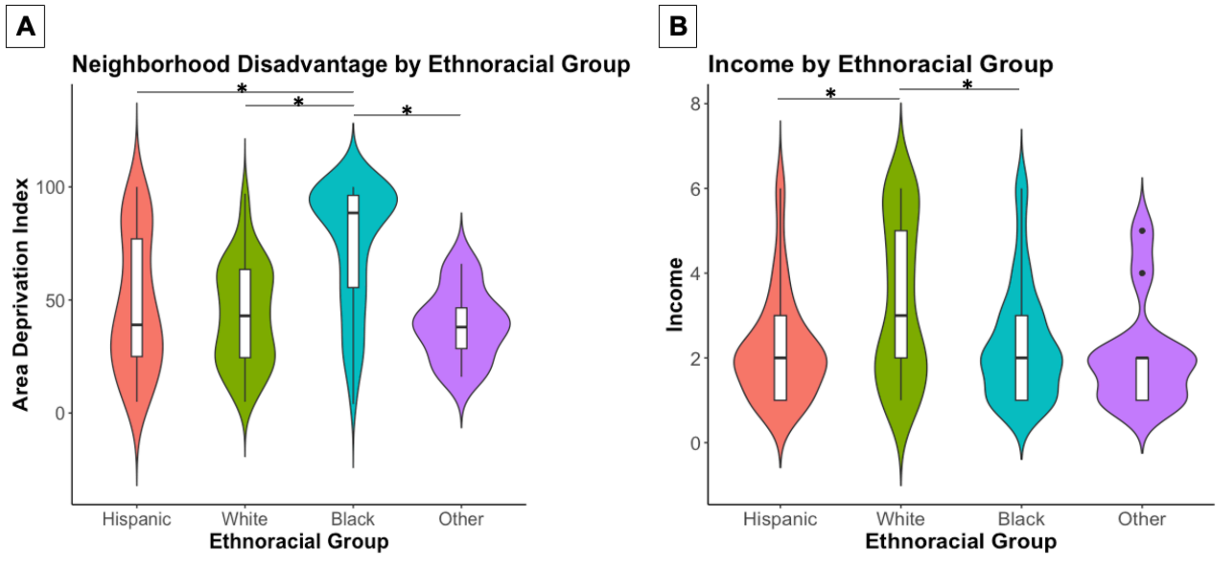

Figure S2. [A] ADI and **[B]** Income significantly differed by ethnoracial group (*ps* < .001; N = 280; threat sample is depicted). ADI ranges from 1-100 (a 1 is indicative of the most advantaged neighborhood relative to all other neighborhoods in the country and a 100 is indicative of the most disadvantaged neighborhood. Income ranged from 1-6 (a 1 is indicative of an income <$19,000 and a 6 is indicative of greater than $100,000). Asterisks depict significant Tukey *post-hoc* tests (*p* < .05 with Hom-Bonferroni correction applied).

© 2023 Webb EK et al. *JAMA Network Open.*

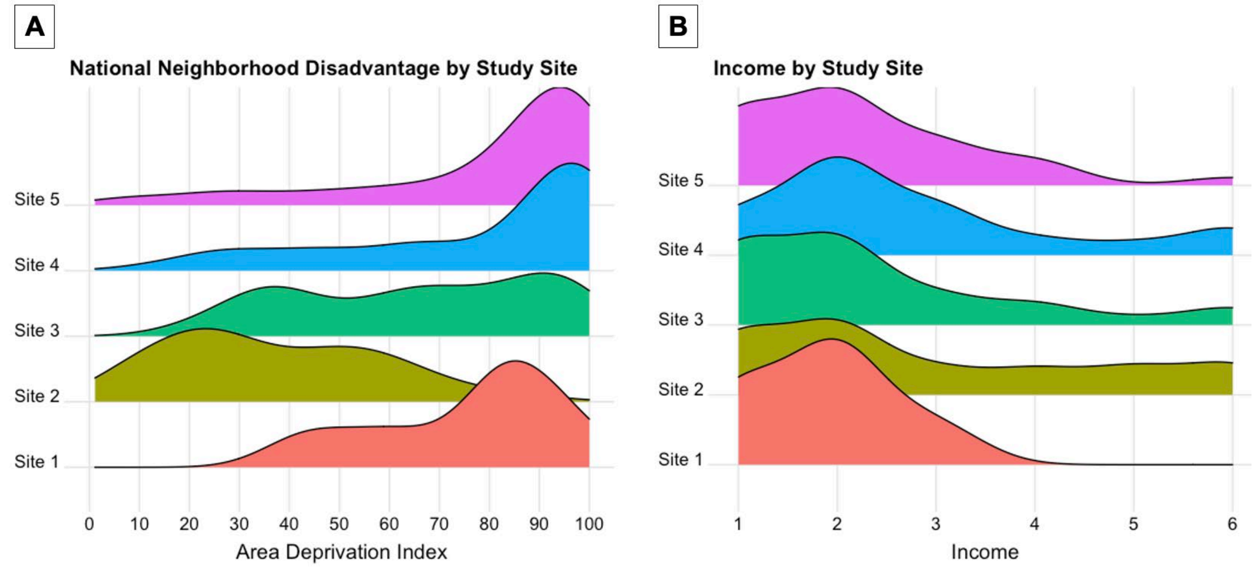

Figure S3. [A] ADI and **[B]** Income significantly differed by site (*ps* < .001; N = 280; threat sample is depicted; *ps* < .05).

© 2023 Webb EK et al. *JAMA Network Open.*

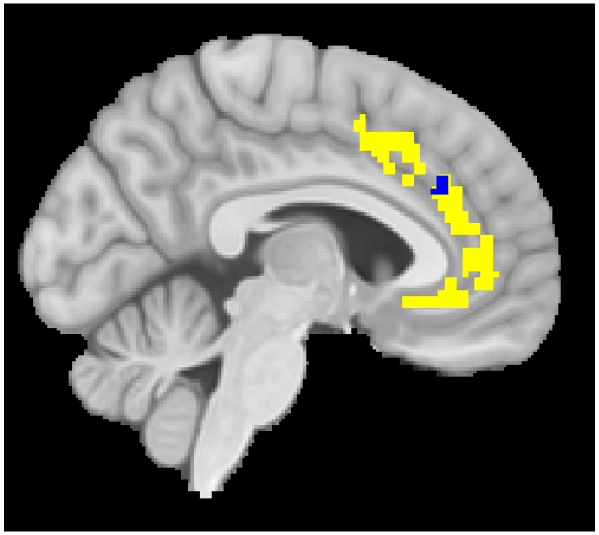

Figure S4. Results of the ROI analysis (N = 280) revealed the significant main effect of neighborhood disadvantage (blue cluster; $k = 12$, X: 4.5, Y: -33.5, Z = 29.5) was in the caudal section of the ACC seed (yellow).

© 2023 Webb EK et al. *JAMA Network Open.*

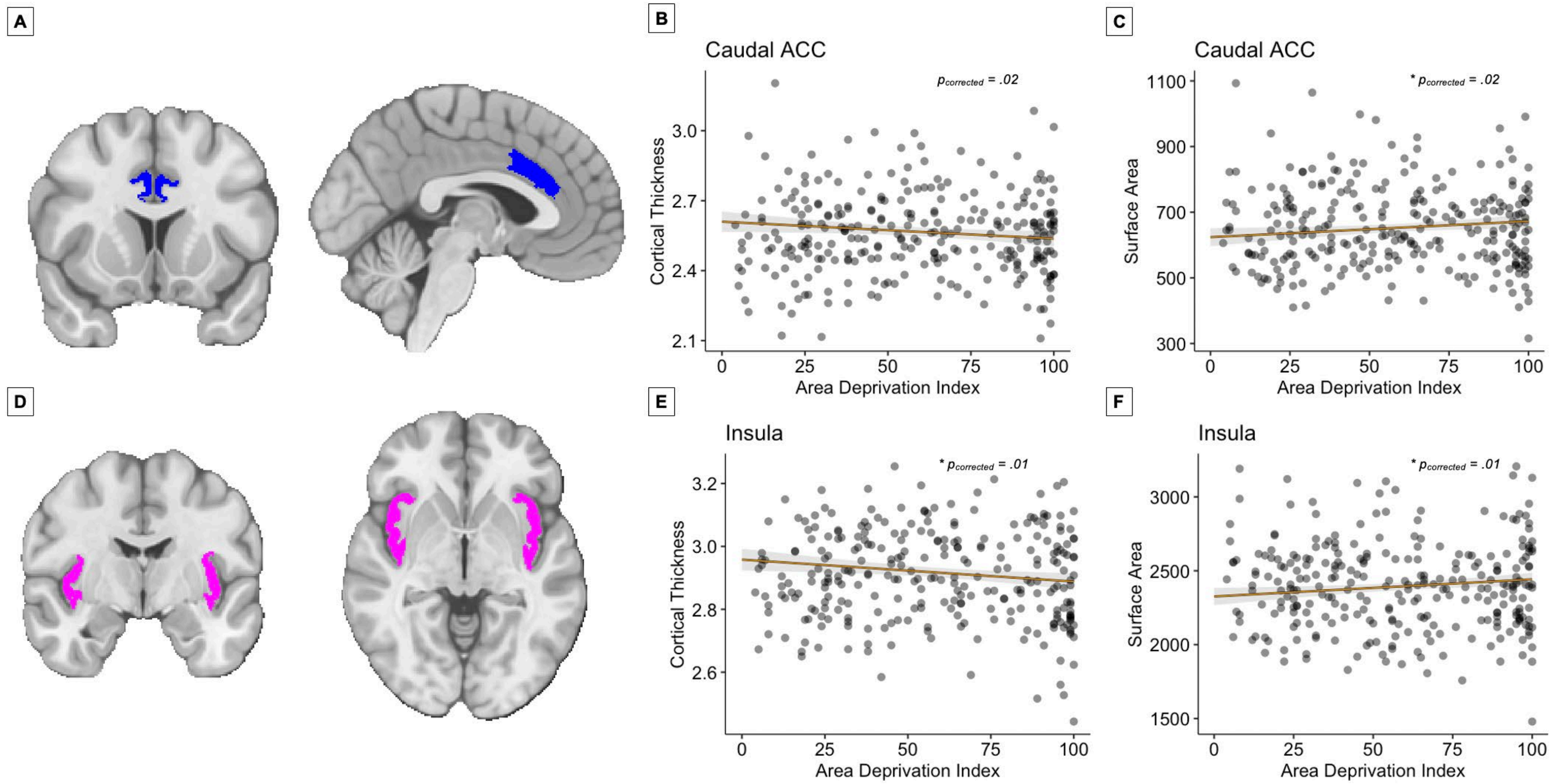

Figure S5. Higher ADI rankings were associated with lower caudal anterior cingulate cortex **[A]** cortical thickness **[B]** and higher **[C]** surface area after adjusting for income, lifetime trauma, sex, age, and total intracranial volume ($N = 280$). Greater neighborhood disadvantage was also associated with lower insula **[D]** cortical thickness **[E]** and higher **[F]** surface area after adjusting for covariates. These are marginal effects plots depicting predicted values (orange regression line) for cortical thickness and surface area at each ADI ranking (shaded line: 95% confidence interval for the marginal effects; datapoints: observed data).

© 2023 Webb EK et al. *JAMA Network Open.*

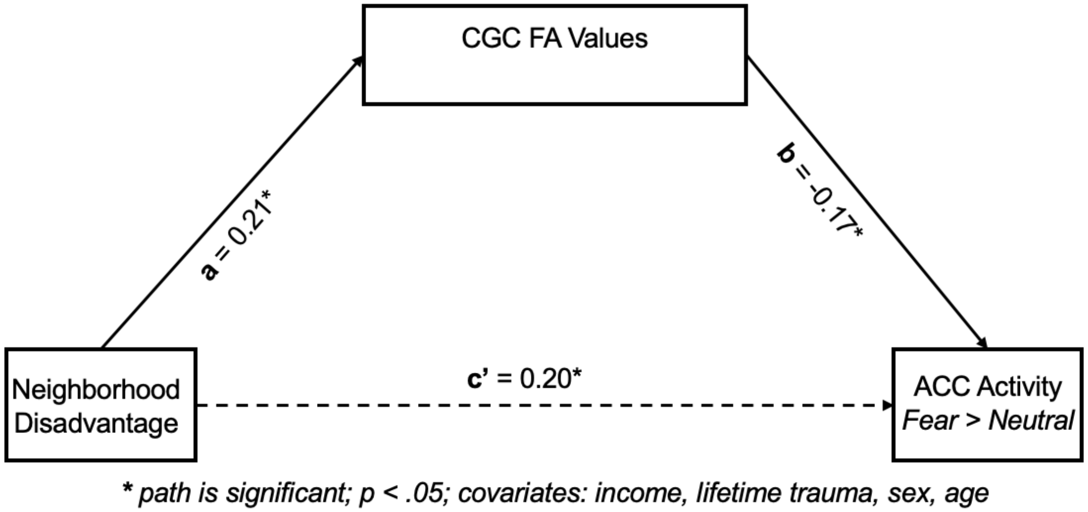

Figure S6. An initial simple mediation model (i.e., no moderator) revealed CGC FA values mediated the relationship between neighborhood disadvantage and ACC reactivity to threat (N = 280). Coefficients are standardized.

© 2023 Webb EK et al. *JAMA Network Open.*

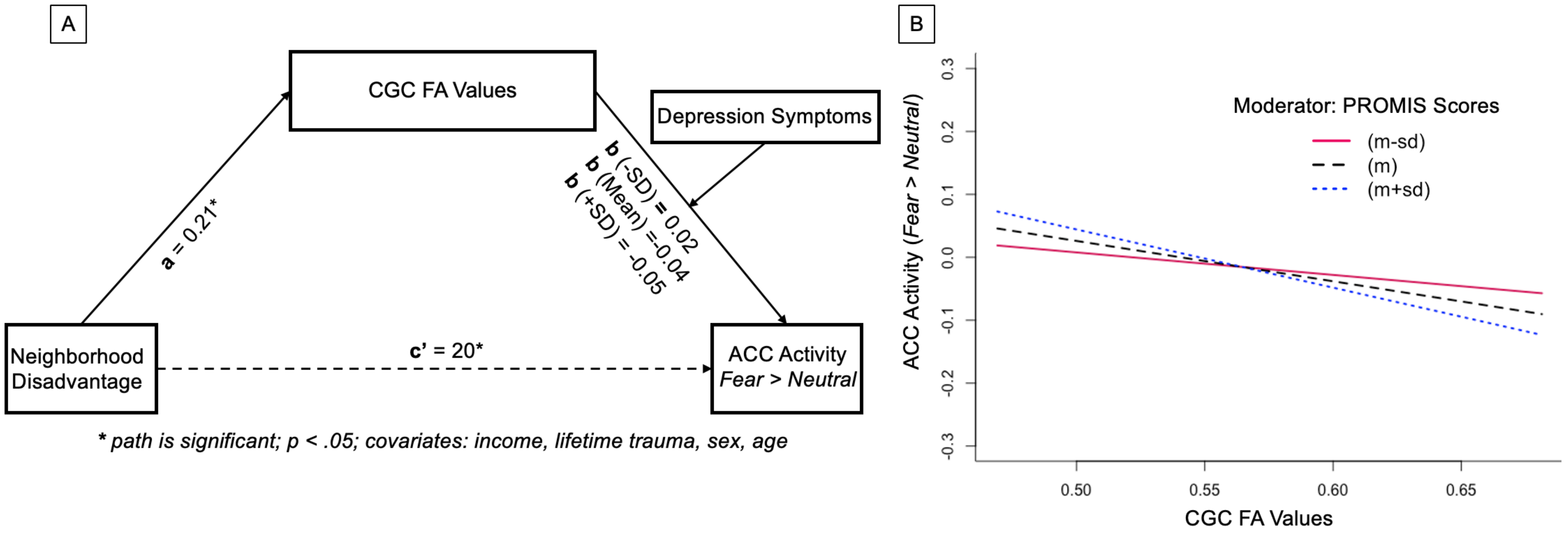

Figure S7. [A] A moderated mediation model revealed depression symptoms did not moderate the association between white matter tract integrity and threat activity. **[B]** Conditional indirect effects of ADI and ACC activity to fearful versus neutral faces via CGC FA values did not significantly differ at higher or lower scores of depression symptoms (*ps* > .05). Coefficients are standardized.

© 2023 Webb EK et al. *JAMA Network Open.*

eReferences

1. American Psychiatric Association AP, Association AP. Diagnostic and statistical manual of mental disorders: DSM-5. Published online 2013.
2. Weathers FW, Litz BT, Keane TM, Palmieri PA, Marx BP, Schnurr PP. The ptsd checklist for dsm-5 (pcl-5). *Scale Available Natl Cent PTSD Www Ptsd Va Gov*. 2013;10(4):206.
3. Pilkonis PA, Choi SW, Reise SP, et al. Item banks for measuring emotional distress from the Patient-Reported Outcomes Measurement Information System (PROMIS®): depression, anxiety, and anger. *Assessment*. 2011;18(3):263-283.
4. Delgado MR, Locke HM, Stenger VA, Fiez JA. Dorsal striatum responses to reward and punishment: effects of valence and magnitude manipulations. *Cogn Affect Behav Neurosci*. 2003;3(1):27-38.
5. Esteban O, Blair R, Markiewicz CJ, et al. poldracklab/fmriprep: 1.0. 0-rc5. *Geneva Zenedo*. Published online 2017.
6. Esteban O, Markiewicz CJ, Blair RW, et al. fMRIPrep: a robust preprocessing pipeline for functional MRI. *Nat Methods*. 2019;16(1):111-116.
7. Tustison NJ, Avants BB, Cook PA, et al. N4ITK: improved N3 bias correction. *IEEE Trans Med Imaging*. 2010;29(6):1310-1320. doi:10.1109/TMI.2010.2046908
8. Fischl B. FreeSurfer. *Neuroimage*. 2012;62(2):774-781.
9. Fonov V, Evans AC, Botteron K, et al. Unbiased average age-appropriate atlases for pediatric studies. *NeuroImage*. 2011;54(1):313-327. doi:10.1016/j.neuroimage.2010.07.033
10. Avants BB, Tustison N, Johnson H. Advanced Normalization Tools (ANTS).

© 2023 Webb EK et al. *JAMA Network Open.*

11. Pruim RHR, Mennes M, van Rooij D, Llera A, Buitelaar JK, Beckmann CF. ICA-AROMA: A robust ICA-based strategy for removing motion artifacts from fMRI data. *NeuroImage*. 2015;112:267-277. doi:10.1016/j.neuroimage.2015.02.064

12. Stevens JS, Harnett NG, Lebois LAM, et al. Brain-Based Biotypes of Psychiatric Vulnerability in the Acute Aftermath of Trauma. *Am J Psychiatry*. 2021;178(11):1037-1049. doi:10.1176/appi.ajp.2021.20101526

13. Harnett NG, Finegold KE, Lebois LAM, et al. Structural covariance of the ventral visual stream predicts posttraumatic intrusion and nightmare symptoms: a multivariate data fusion analysis. *Transl Psychiatry*. 2022;12(1):1-13. doi:10.1038/s41398-022-02085-8

14. Esteban O, Birman D, Schaer M, Koyejo OO, Poldrack RA, Gorgolewski KJ. MRIQC: Advancing the automatic prediction of image quality in MRI from unseen sites. *PLOS ONE*. 2017;12(9):e0184661. doi:10.1371/journal.pone.0184661

15. Power JD, Barnes KA, Snyder AZ, Schlaggar BL, Petersen SE. Spurious but systematic correlations in functional connectivity MRI networks arise from subject motion. *Neuroimage*. 2012;59(3):2142-2154. doi:10.1016/j.neuroimage.2011.10.018

16. Harnett NG, Dumornay NM, Delity M, et al. Prior differences in previous trauma exposure primarily drive the observed racial/ethnic differences in posttrauma depression and anxiety following a recent trauma. *Psychol Med*. 2023;53(6):2553-2562. doi:10.1017/S0033291721004475

© 2023 Webb EK et al. *JAMA Network Open.*
