# Supplementary material for: Neighborhood Disadvantage and Neural Correlates of Threat and Reward Processing in Survivors of Recent Trauma
Source: JAMA Netw Open. 2023 Sep 18;6(9):e2334483. doi: 10.1001/jamanetworkopen.2023.34483 (PMC10507487; doi:10.1001/jamanetworkopen.2023.34483)
Supplement: Supplement 2. — Data Sharing Statement [file jamanetwopen-e2334483-s002.pdf]

Data Sharing Statement

Webb. Neighborhood Disadvantage and Neural Correlates of Threat and Reward Processing in Survivors of Recent Trauma. *JAMA Netw Open*. Published online September 18, 2023. doi:10.1001/jamanetworkopen.2023.34483

Data
Data available: Yes
Data types: Deidentified participant data
How to access data: Data is available through National Institute of Mental Health (NIMH) Data Archive (NDA). Dataset identifier(s): NIMH Data Archive Digital Object Identifier (DOI) 10.15154/1528773.
When available: With publication

Supporting Documents
Document types: None

Additional Information
Who can access the data: Individuals can access this data without researchers approval through through National Institute of Mental Health (NIMH) Data Archive (NDA). Dataset identifier(s): NIMH Data Archive Digital Object Identifier (DOI) 10.15154/1528773.
Types of analyses: Individuals can access this data without researchers approval through through National Institute of Mental Health (NIMH) Data Archive (NDA). Dataset identifier(s): NIMH Data Archive Digital Object Identifier (DOI) 10.15154/1528773.
Mechanisms of data availability: Individuals can access this data without researchers approval through through National Institute of Mental Health (NIMH) Data Archive (NDA). Dataset identifier(s): NIMH Data Archive Digital Object Identifier (DOI) 10.15154/1528773.
